# Reversing Threat to Safety: Incongruence of Facial Emotions and Instructed Threat Modulates Conscious Perception but Not Physiological Responding

**DOI:** 10.3389/fpsyg.2019.02091

**Published:** 2019-09-13

**Authors:** Florian Bublatzky, Martin Riemer, Pedro Guerra

**Affiliations:** ^1^Department of Psychosomatic Medicine and Psychotherapy, Central Institute of Mental Health Mannheim, Medical Faculty Mannheim/Heidelberg University, Mannheim, Germany; ^2^Department of Psychology, School of Social Sciences, University of Mannheim, Mannheim, Germany; ^3^Aging & Cognition Research Group, German Center for Neurodegenerative Diseases (DZNE), Magdeburg, Germany; ^4^Faculty for Behavioural and Social Sciences, University of Groningen, Groningen, Netherlands; ^5^Department of Personality, University of Granada, Granada, Spain

**Keywords:** reversal learning, emotional facial expression, threat-of-shock, startle reflex, social learning

## Abstract

Facial expressions inform about other peoples’ emotion and motivation and thus are central for social communication. However, the meaning of facial expressions may change depending on what we have learned about the related consequences. For instance, a smile might easily become threatening when displayed by a person who is known to be dangerous. The present study examined the malleability of emotional facial valence by means of social learning. To this end, facial expressions served as cues for verbally instructed threat-of-shock or safety (e.g., “happy faces cue shocks”). Moreover, reversal instructions tested the flexibility of threat/safety associations (e.g., “now happy faces cue safety”). Throughout the experiment, happy, neutral, and angry facial expressions were presented and auditory startle probes elicited defensive reflex activity. Results show that self-reported ratings and physiological reactions to threat/safety cues dissociate. Regarding threat and valence ratings, happy facial expressions tended to be more resistant becoming a threat cue, and angry faces remain threatening even when instructed as safety cue. For physiological response systems, however, we observed threat-potentiated startle reflex and enhanced skin conductance responses for threat compared to safety cues regardless of whether threat was cued by happy or angry faces. Thus, the incongruity of visual and verbal threat/safety information modulates conscious perception, but not the activation of physiological response systems. These results show that verbal instructions can readily overwrite the intrinsic meaning of facial emotions, with clear benefits for social communication as learning and anticipation of threat and safety readjusted to accurately track environmental changes.

## Introduction

Emotional facial expressions – signaling anger, fear or happiness of the current interaction partners – are essential to organize social behavior. Although the processing of emotional facial expressions has been suggested to be evolutionary prepared fostering appropriate responding (e.g., fight or flight), the meaning of facial emotions can readily change depending on learning and explicit knowledge about related consequences. For instance, a dangerous person smiling at you might be much more threatening than a good friend looking angry. Such information about threat and safety contingencies, acquired through verbal communication (e.g., statements like “this is dangerous”), has been shown to consistently activate defensive response systems (e.g., threat-potentiated startle reflex; Grillon et al., 1991; Bradley et al., 2005; Bublatzky et al., 2013). However, the malleability of emotional facial valence and person perception by means of social learning through verbal instructions is less understood (Bublatzky et al., 2018).

Much recent research in humans examined facial expressions as a key aspect of non-verbal communication. With clear benefits for adequate interaction behavior, facial emotions guide perceptual processing and psychophysiological responding in social situations. For instance, expressions of anger or happiness have been shown to be associated with preferential neural processing relative to neutral faces (e.g., in the amygdala or temporo-occipital cortex; Phelps and LeDoux, 2005; Adolphs, 2008; Bublatzky et al., 2014b, 2017b; Schindler et al., 2019). This processing advantage presumably sets the stage for overt behaviors such as speeded response times (Öhman et al., 2001; Craig et al., 2014) or decisions to approach or avoid a feared stimulus or situation (e.g., Bublatzky et al., 2017a; Pittig et al., 2018). Regarding the activity of the somatic and autonomic nervous system while viewing facial emotions, however, result patterns are mixed. For instance, some studies show potentiated startle reflex to fearful and angry faces (Springer et al., 2007; Anokhin and Golosheykin, 2010), which can vary with the gender of a face (Hess et al., 2007). Other studies reported startle potentiation based on stimulus arousal (i.e., angry and happy versus neutral faces; Bublatzky and Alpers, 2017) but only in highly social anxious participants (Garner et al., 2011; Wangelin et al., 2012). Taken together, these response patterns presumably reflect the functionality of basic motivational circuits that guide approach or withdrawal in survival-relevant situations (Lang and Bradley, 2010), however, less is understood regarding social situations.

Verbal communication is highly effective to inform others about future benefits and detriments. Similar to visual signals of danger, verbal threat instructions have been shown to enhance perceptual processing (Bublatzky et al., 2010; Mechias et al., 2010; Bublatzky and Schupp, 2012) and prime the activation of physiological defense mechanisms (e.g., threat-potentiated startle reflex; Grillon et al., 1991; Bradley et al., 2005; Bublatzky et al., 2013). Interestingly, learning through verbal instructions does not need to be proved by first-hand experiences. Whereas the effects of instructed threat can be very resistant against extinction learning (i.e., even across repeated test days; Bublatzky et al., 2013, 2014a), such associations can be flexibly changed by means of reversal instructions (Schiller et al., 2008; Costa et al., 2015; Atlas and Phelps, 2018). For instance, reversal instructions readily attenuated defensive activation when the meaning of a threat cue was changed to cueing safety (Costa et al., 2015; Mertens and De Houwer, 2016). Thus, verbal information can flexibly establish and reverse previously acquired threat and safety associations; whether this reversal learning process depends on evolutionary prepared mechanisms in face and person perception is not well understood (e.g., Mallan et al., 2009; Rowles et al., 2012).

The present study examined the interaction of visual and verbal affective information by means of facial emotions and threat/safety instructions. In a between-group design, happy and angry facial expressions served as cues for instructed threat-of-shock or safety (e.g., happy faces cue threat and angry faces cue safety, or vice versa). Following this, a second reversal block changed the previously acquired threat/safety associations, in that now only neutral faces cued threat-of-shock. Using a similar design in a companion study (Bublatzky et al., 2018), we could show that the acquisition of threat associations was highly effective regardless of which facial emotion cued threat or safety (in Block 1). Moreover, reversal instructions readily changed threat/safety associations linked to happy and angry facial expression (in Block 2). However, because the reversed threat cues were always emotional expressions (either angry or happy; Bublatzky et al., 2018), testing the stability of threat effects after reversal was confounded by the facial emotions. Another interesting finding showed that, regardless of which emotion cued threat, reversal effects were more stable in trait and socially anxious participants.

Based on these findings, we derived several hypotheses for the present study. First, regarding the initial acquisition of threat and safety associations, we expected pronounced activation of the autonomic and somatic nervous systems for threat relative to safety cues. This defensive response pattern has been observed previously for neutral objects or affective scenes cueing threat-of-shock (Bradley et al., 2005; Costa et al., 2015). Moreover, replicating our previous findings using facial expressions (Bublatzky et al., 2018), potentiated defensive startle reflex, enhanced skin conductance responses, and HR-deceleration are predicted regardless of whether happy or angry faces served as instructed threat cue (in Block 1).

Second, the *a priori* valence (i.e., intrinsic affective meaning) of an emotional facial expression was expected to influence the stability of instructed threat effects. This hypothesis relates to previous research that tested visual facial information as an “evolutionary prepared” stimulus type similar to pictures of snakes and spiders (e.g., Seligman, 1971; Lipp and Edwards, 2002; Berdica et al., 2018). For instance, Rowles et al. (2012) observed more persistent threat effects when angry (but not happy) facial expressions served as conditioned threat cue in a Pavlovian fear conditioning experiment. A similar resistance to extinction of threat-associations has been observed for out- compared to in-group faces (Olsson et al., 2005; Mallan et al., 2009) using skin conductance responses as the key dependent variable. For reversal learning – reflecting the transfer of threat-value from one stimulus to another – we analogously hypothesized persistent threat-associations when threat was previously acquired to potentially threatening faces. Specifically, given the flexible reversal of threat and safety contingencies (Costa et al., 2015; Atlas and Phelps, 2018), threat-of-shock transferred from angry to neutral facial expressions should be associated with pronounced threat-potentiated startle reflex and elevated sympathetic system activation following the reversal instruction (in Block 2).

Third, building upon the notion of evolutionary prepared stimuli, an alternative hypothesis regards the capability of happy facial expressions to acquire safety-associations (Hornstein et al., 2016; Hornstein and Eisenberger, 2018). According to this notion, a smiling face, which previously cued threat, might be readily learned as a safety signal. Consequently, the transfer of threat-associations from happy to neutral faces might lead to pronounced defensive responding (i.e., threat-potentiated startle reflex, SCRs and HR deceleration). Alternatively, fourth, threat learning might vary as a function of the incongruence between intrinsic facial valence and explicitly instructed threat or safety contingencies. For instance, incongruent facial emotions and affective sounds led to increased activation in areas involved in conflict monitoring (e.g., cingulate cortex and superior frontal cortex; Müller et al., 2011), and selective processing of pleasant picture materials has been found in a context of instructed threat (Bublatzky et al., 2010). Finally, correlational analyses were conducted to replicate our previous finding of persistent threat effects after reversal instructions in more trait and socially anxious participants (Bublatzky et al., 2018).

## Materials and Methods

### Participants

Sample size was chosen similar to previous research using facial expressions and instructed threat manipulations (e.g., Bradley et al., 2005; Grillon and Charney, 2011; Bublatzky et al., 2018). Moreover, statistical estimations with G^∗^Power (Faul et al., 2009), indicated that at least *N* = 36 was required to detect relevant effects at a medium effect size (*f* = 0.2) and power (1−β = 0.8). Forty healthy participants (five males) were recruited from the students of the University of Mannheim. Age was between 19 and 29 (*M* = 22.1, *SD* = 2.7), and participants were within the normal range of state and trait anxiety (STAI, *M* = 36.0 and 38.2, *SD* = 8.6 and 9.9), social anxiety (SPIN, *M* = 14.7, *SD* = 7.8), and depression (BDI, *M* = 6.8, *SD* = 7.5). Exclusion criteria were acute or chronic medical or psychiatric disorders, or the previous participation in an experiment with the administration of electric shocks. All participants provided informed and written consent to the study procedure, which was approved by the local ethics committee. Participants received course credits for participation.

Participants were assigned to one of two experimental groups, which were differently instructed regarding threat-of-shock and safety. Depending on the group, either angry or happy facial expressions were introduced as threat cues in the first block (e.g., angry faces cued threat-of-shock while happy and neutral faces cued safety). In a second block, threat- and safety associations were partially reversed in that neutral facial expressions served as threat cues for both groups, whereas happy and angry faces cued safety during this reversal block. Accordingly, two threat-sequences were tested (i.e., angry-neutral and happy-neutral group)^1^. Both groups were verbally instructed that ‘unpleasant, but not painful electric shocks, might occur when a particular facial expression was presented (e.g., “all angry faces indicate threat of electric shock”), though not when other facial expressions were visible (e.g., “all happy and neutral faces indicate safety”).

### Stimulus Materials and Presentation

**FIGURE 1:**
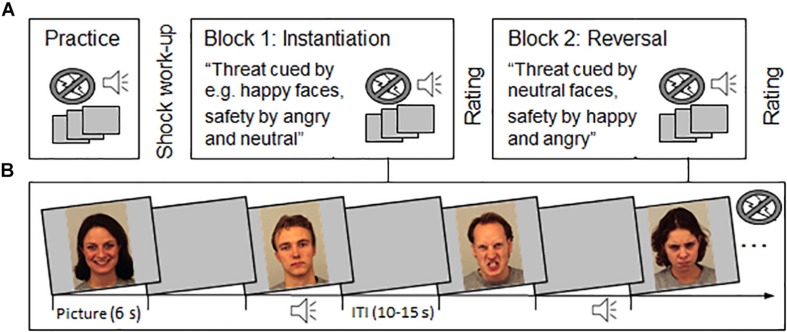
Schematic illustration of the experimental procedure **(A)** and stimulus presentation **(B)**. **(A)** A brief practice run and shock work-up procedure preceded the experiment. In the first experimental block (instantiation), participants were verbally instructed that one particular emotional facial expression signals threat-of-shock (e.g., happy) or safety (e.g., angry and neutral faces). The second experimental block (reversal), started with a verbal reversal instruction stating that now threat and safety contingencies are reversed. Now neutral faces cued shock threat in both experimental groups, and happy and angry faces signaled safety in Block 2. The order in which facial expressions cued threat (happy–neutral or angry–neutral) was tested in two groups of each *N* = 20. After each block, threat and safety cues were rated regarding valence, arousal, and perceived threat. **(B)** During the experimental blocks, happy, neutral and angry face pictures were presented (each 6 s) with a variable inter-trial interval (ITI, 10 to 15 s). In total, 24 pictures were presented together with an auditory startle probe (and six ITI startles), which were equally distributed across experimental conditions. No shocks were presented during the experiment. Example pictures are taken from the KDEF with permission (identifiers: af01has, am08nes, am10ans, and af20ans; see Lundqvist et al., 1998; http://kdef.se/home/aboutKDEF.html).

Face pictures of 16 actors^2^ (1024 × 768 pixels) displaying happy, neutral, and angry facial expressions were chosen from the Karolinska Directed Emotional Faces (KDEF; Lundqvist et al., 1998). All pictures were presented once for 6 s followed by an inter-trial interval (ITI) ranging from 10 to 15 s to allow response recovery (see Figure 1). To provoke the defensive eye-blink startle reflex, half of the picture trials were accompanied with auditory startle probes (white noise 105 dB, 50 ms). Startle probes were presented between 4 to 5.5 s after picture onset and the mean distance between probes was 28.8 s. Six additional probes were presented during the ITI to prevent predictability of the startle probe presentation.

The total set of 48 picture trials, including the 24 picture-startle trials, was evenly distributed across two experimental blocks (instantiation and reversal) and facial expressions (happy, neutral, angry). Thus, per participant, 4 picture-startle trials contributed to each experimental condition. All participants viewed different picture sequences that were pseudorandom with regard to the order of face actors and facial expressions. The restriction criteria were no immediate repetition of the same actor and no more than three repetitions of the same emotional expression.

### Procedure

After completing anxiety and depression questionnaires (State-Trait Anxiety Inventory, Spielberger, 2010; Social Phobia Inventory, Connor et al., 2000; Beck Depression Inventory, Beck et al., 1988), participants were seated in a sound attenuated and air conditioned room. Sensors for physiological recordings and headphones for startle probe presentation were attached. Furthermore, an electric stimulation electrode was placed at the right upper arm, and a brief shock work-up procedure was carried out to ensure the credibility of the threat instruction (cf. Riemer et al., 2015; Bublatzky et al., 2017a). To this end, participants received up to ten shocks with increasing intensity (max. 10 mA, 100 ms) until shock intensity was rated as “maximally unpleasant but not yet painful”. Participants were then told that the electric shocks given during the experiment would be equally intense as the most unpleasant test stimulus. Twelve practice trials (with eight startle probes; not analyzed) preceded the experiment to allow initial response habituation of the startle reflex and to familiarize participants with the picture and sound presentation procedure.

Main instructions regarding threat and safety associations were given and, depending on the experimental group, half of the participants started with either angry or happy facial expressions as threat cues. Following a brief break in the middle of the experiment, threat associations were reversed in that for both groups now neutral faces served as threat cues (i.e., angry-neutral or happy-neutral group). During the break, and at the end of the experiment, participants rated the perceived threat, valence, and arousal of the facial expressions using a visual analog scale ranging from *not at all* to *highly threatening* (1 to 10) and the Self-Assessment Manikin (SAM; Bradley and Lang, 1994). During both experimental blocks, no shocks were administered.

### Data Recording and Reduction

Physiological measures were recorded with a vAmp amplifier (BrainProducts, Munich, Germany). For measuring the defensive eye-blink startle reflex, two miniature Ag/AgCl electrodes assessed EMG activity of the left orbicularis muscle. The signal was acquired at a 1000 Hz sampling rate and frequencies below 28 Hz and above 500 Hz were canceled out by means of a band-pass filter (24 dB/octave roll-off). The raw EMG was then rectified and smoothed by using a moving average procedure (50 ms) in VisionAnalyzer 2.1 (BrainProducts). An automated procedure served to score startle responses as the maximum peak in the 21–150 ms time window following auditory startle probes. Startle amplitudes were calculated as the maximum peak relative to the mean baseline period (50 ms) preceding the startle response time window (i.e., −30 ms to + 20 ms around the startle probe; Blumenthal et al., 2005). Startle trials showing clear movement artifacts, excessive baseline activity or non-responses were excluded (i.e., peaks not exceeding 4 SD from mean baseline activity; overall 2.4% of the trials). Within individuals amplitudes were standardized across trials and transformed to T scores [(amplitude – mean amplitude)/SD ^∗^ 10 + 50].

As an indicator of enhanced activity of the sympathetic system, skin conductance responses (SCRs) were recorded using Ag/AgCl electrodes (constant voltage of 0.5 V; 20 Hz sampling rate) placed at the hypothenar eminence of the left hand. Noise and slow frequency changes were removed using a 2 Hz FIR low- and a 0.05 Hz high-pass filter. SCRs to picture onset were scored as the maximum peak within a time interval of 1 to 6 s relative to a 1 s pre-picture period. Zero-response detection was based on a minimum threshold of 0.02 μS, and range and distribution correction were applied within each participant [square root (response/maximum response)].

As an indicator of the combined activity of sympathetic and parasympathetic systems, phasic heart rate changes were derived from the electrocardiogram (ECG) recorded at lead II and at a 1000 Hz sampling rate. Frequencies below 0.1 Hz and above 13 Hz were filtered out. Heart rate was determined by averaging across each half-second and subtracting the same activity from the 2 s prior to the picture onset (Bradley et al., 2005).

### Data Analysis

Mean amplitudes of the rating data (perceived threat, valence, and arousal), as well as startle reflex and skin conductance responses were analyzed with (2 × 2) × 2 repeated measures ANOVAs. Within-subject factors were Instruction (threat vs. safety) and Block (first instantiation vs. second reversal), as well as Group (angry-neutral vs. happy-neutral) serving as a between-subject factor^3^. The group factor coded the block-sequence in which the facial expressions cued threat or safety. In the instantiation Block 1, threat-of-shock was signaled by either angry or happy faces (angry-neutral or happy-neutral group), and neutral faces cued threat for both groups in the reversal Block 2. Examining the impact of *a priori* valence of facial expressions on the instantiation and reversal of threat-contingencies, follow-up comparisons focused on each group separately (angry-neutral and happy-neutral group). For phasic heart rate changes, an additional factor Time (12 time bins) was used to compare half-second changes after picture onset. To examine the impact of interindividual differences in social- and trait-anxiety on the defensive startle reflex (cf. Bublatzky et al., 2018), covariation and correlational analyses were conducted with questionnaire scores. To quantify threat effects, difference scores (threat minus safety) were calculated for each block separately.

In addition, we conducted Bayesian analyses to provide more information about non-significant effects of our key hypothesis (i.e., estimates of the probability of the null- relative to the alternative hypothesis; Kass and Raftery, 1995). Here, a focus is set on the of-interest interaction between threat/safety instructions, instantiation and reversal learning, and the facial expressions serving as threat/safety cues (i.e., Instruction × Block × Group). Bayes factors (BF) were estimated for all relevant models (Instruction, Block, Instruction + Block, Instruction + Block + Order^∗^Order, and so on; see Table 1) using Monte-Carlo sampling 10000 iterations and default prior scaling factors (for fixed effects = 0.5, random effects = 1; Rouder et al., 2012) using the R based software package JASP (Morey et al., 2015; JASP Team, 2018). BF inclusion scores (BF_Incl_) are reported and inform about how much the inclusion of one factor (e.g., Instruction, averaged over all models that include this factor) is supported by the data, compared to all other models (including the null-model). A value of 1 suggests that both null and alternative hypotheses are equally probable with the data at hand, while values below (above) 1 indicate that the data are more (less) likely under the null relative to the alternative hypothesis. For instance, a BF < 0.333 means that the data is at least three times more likely under the null relative to the alternative hypotheses (and vice versa for BF > 3).

**TABLE 1:** Bayes factors (BF_incl_) of the selected models compared to all models without this factor for the different dependent measures.

**Model**	**BF_Inclusion_:**	**Startle**	**SCR**	**HR**	**Threat**	**Valence**	**Arousal**
Block		3.217^∗^10^15^	49.064	0.257	63.68	31.93	1.743
Instruction		3.247^∗^10^9^	252.357	3.471	89376.17	5242.31	3.587^∗^10^9^
Order		0.162	0.273	0.146	99.98	214.69	0.245
Block × Instruction		7.609	1.215	0.212	316.48	153.00	5.177
Block × Order		0.289	0.317	0.117	308.93	152.01	0.283
Instruction × Order		0.178	0.313	0.240	334.46	175.63	0.189
Block × Instruction × Order		0.125	0.072	0.027	2507.83	1169.98	0.119

*Bayes factors (BF_*incl*_) of the selected models compared to all models without this factor for the different dependent measures.*

Greenhouse-Geisser corrections were applied when necessary, 95% confidence intervals (CI) and the partial η^2^ is reported as effect size. Controlling for Type 1 error, Bonferroni correction was applied for *post hoc t*-tests.

## Results

### Self-Report Data

#### Threat Ratings

**FIGURE 2:**
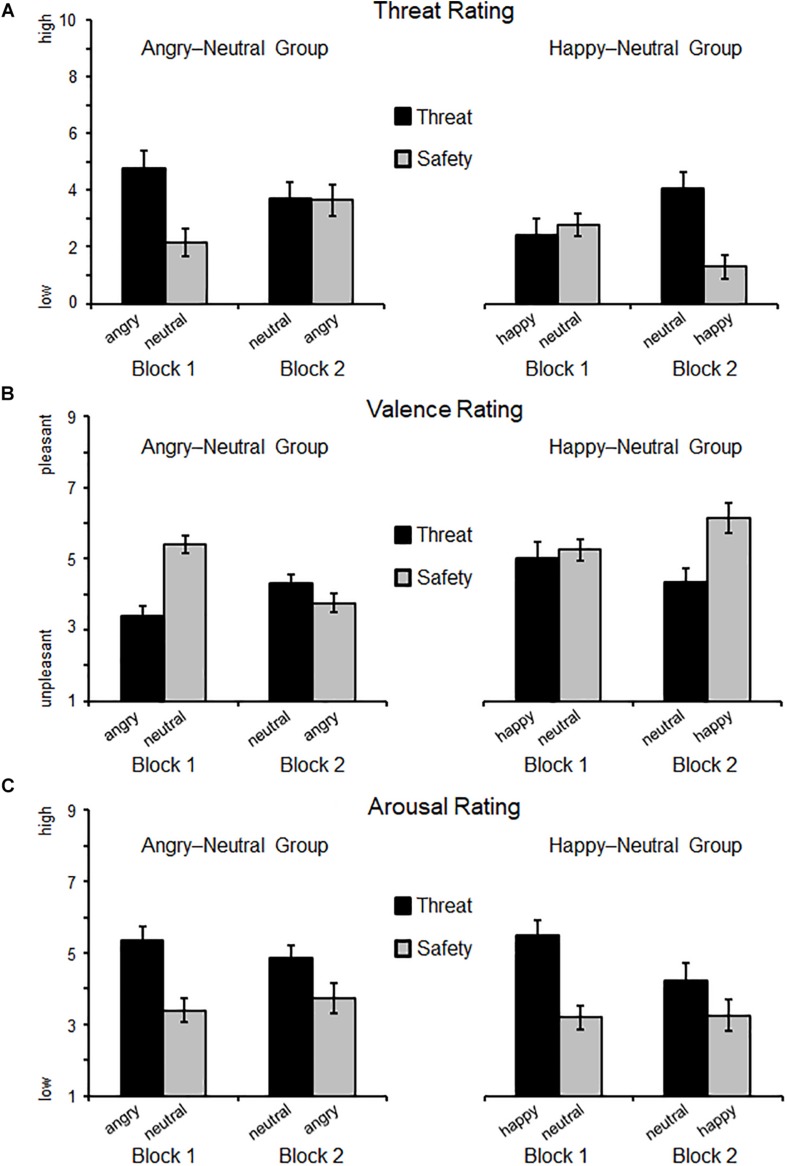
Self-reported threat **(A)**, valence **(B)**, and arousal **(C)** ratings as a function of Block (first, second) and Instruction (threat, safety). Means (SEM) are plotted separately for each group: on the left side, the Angry–Neutral Group started with angry expression as threat cue, and on the right the Happy–Neutral Group with happy faces cueing threat in the first block. For both groups neutral faces served as reversed threat cue in the second block.

Self-reported threat varied as a joint function of Instruction × Block × Group (*F*(1,38) = 25.77, *p* < 0.001, η_p_^2^ = 0.40, BF_incl_ = 2507.83). Follow-up analyses focused on each experimental group separately (see Figure 2A and Table 2 for *M*, *SD*, and 95% CI). For the angry-neutral group, instructed threat cues were rated as more threatening relative to the safety cues (*F*(1,19) = 14.95, *p* = 0.001, η_p_^2^ = 0.44). Whereas no main effect of Block was observable (*F*(1,19) = 0.72, *p* = 0.41, η_p_^2^ = 0.04) a significant interaction Instruction × Block emerged (*F*(1,19) = 20.43, *p* < 0.001, η_p_^2^ = 0.52). For Block 1, augmented threat ratings were observed when angry faces cued threat-of-shock (*p* < 0.001) but no difference was found for neutral expressions cueing threat compared to angry faces cueing safety (Block 2; *p* = 0.92). For the happy-neutral group significant effects emerged for Instruction (*F*(1,19) = 7.85, *p* = 0.011, η_p_^2^ = 0.29) and the interaction Instruction × Block (*F*(1,19) = 10.44, *p* = 0.004, η_p_^2^ = 0.36). This indicates enhanced threat ratings for neutral faces cueing threat compared to happy faces cueing safety in Block 2 (*p* < 0.001) but no differences were observed when happy faces cued shocks in Block 1 (*p* = 0.64). Thus, regarding threat ratings, happy facial expressions seemed to be more resistant to becoming threat cues (relative to neutral safety cues), and angry faces remained threatening even when instructed to signal safety.

**TABLE 2:** Means (*SD*, and 95% CI) for the physiological measures (startle, SCR, and HR) and ratings (valence, arousal, and threat) as a function of Block (first, second), Instruction (threat, safety), and experimental Group (Angry-Neutral, Happy-Neutral).

**Block**	**Instruction**	**Group**	**Startle**			**SCR**			**HR**			**Valence**			**Arousal**			**Threat**		
								
			***M***	***SD***	**95% Cl**	***M***	***SD***	**95% Cl**	***M***	***SD***	**95% Cl**	**M**	***SD***	**95% Cl**	**M**	***SD***	**95% Cl**	**M**	***SD***	**95% Cl**
Block 1	Threat	**Angry**-Neutral	57.55	1.02	[55.42, 59.67]	0.19	0.03	[0.12, 0.26]	−1.3	0.31	[−1.94, −0.65]	3.4	0.26	[2.87, 3.94]	5.35	0.41	[4.5, 6.2]	4.75	0.62	[3.45, 6.05]
		**Happy**-Neutral	58.48	1.32	[55.69, 61.27]	0.25	0.05	[0.15, 0.351	−2.3	0.78	[−3.94, −0.66]	5.0	0.49	[3.97. 6.03]	5.5	0.44	[4.58, 6.42]	2.45	0.57	[1.25, 3.65]
	Safe	Angry-Neutral	50.56	0.72	[49.06, 52.06]	0.11	0.02	[0.06, 0.16]	−0.65	0.32	[−1.32, 0.02]	5.4	0.25	[4.89, 5.91]	3.4	0.32	[2.73, 4.07]	2.15	0.47	[1.16, 3.14]
		Happy-Neutral	52.03	0.89	[50.16, 53.9]	0.13	0.03	[0.08, 0.19]	−0.70	0.33	[−1.4, 0.00]	5.25	0.31	[4.61,5.89]	3.2	0.33	[2.51,3.89]	2.8	0.40	[1.97, 3.63]
Block 2	Threat	Angry-**Neutral**	49.38	1.15	[46.98, 51.78]	0.12	0.03	[0.06, 0.17]	−0.90	0.36	[−1.65, −0.16]	4.3	0.26	[3.75, 4.85]	4.85	0.38	[4.06, 5.64]	3.7	0.56	[2.53, 4.871
		Happy-**Neutral**	47.84	1.05	[45.62, 50.06]	0.14	0.05	[0.05, 0.24]	−1.31	0.50	[−2.36, −0.26]	4.35	0.39	[3.54,5.16]	4.25	0.48	[3.24, 5.26]	4.05	0.57	[2.85, 5.25]
	Safe	Angry-Neutral	45.22	0.62	[43.93, 46.51]	0.08	0.02	[0.04, 0.12]	−0.82	0.52	[−1.92, 0.28]	3.75	0.27	[3.18, 4.32]	3.75	0.42	[2.87, 4.63]	3.65	0.56	[2.53, 4.871
		Happy-Neutral	45.24	0.72	[43.72, 46.76]	0.07	0.02	[0.02, 0.12]	−0.06	0.39	[−0.88, 0.75]	6.15	0.43	[5.25, 7.05]	3.25	0.44	[2.33, 4.17]	1.3	0.42	[0.41, 2.19]

#### Valence Ratings

A significant three-way interaction (Instruction × Block × Group, *F*(1,38) = 14.86, *p* < 0.001, η_p_^2^ = 0.28, BF_incl_ = 1169.98) emerged for valence ratings. Separate analyses for the angry-neutral group (see Figure 2B) revealed that threat relative to safety cues were rated as more unpleasant (*F*(1,19) = 9.87, *p* < 0.01, η_p_^2^ = 0.34) and overall unpleasantness increased across blocks (*F*(1,19) = 5.98, *p* < 0.05, η_p_^2^ = 0.24). Moreover, the interaction Instruction × Block was significant (*F*(1,19) = 14.98, *p* = 0.001, η_p_^2^ = 0.44) showing pronounced unpleasantness for angry faces cueing threat in Block 1 (*p* < 0.001) but comparable valence ratings for neutral faces signaling threat and angry faces cueing safety in Block 2 (*p* = 0.212). For the happy-neutral group, instructed threat effects emerged (*F*(1,19) = 14.67, *p* = 0.001, η_p_^2^ = 0.44) but no main effect of Block (*F*(1,19) = 0.30, *p* = 0.59, η_p_^2^ = 0.02) nor an interaction Instruction × Block (*F*(1,19) = 3.45, *p* = 0.079, η_p_^2^ = 0.15). Follow-up tests revealed no differences for happy faces cueing threat compared to neutral safety cues in Block 1 (*p* = 0.62) but more pleasantness for happy faces cueing safety compared to neutral faces cueing threat-of-shock in Block 2 (*p* = 0.002). For the valence ratings, happy faces cueing threat did not become more unpleasant than neutral faces (signaling safety), and angry faces signaling safety were rated as unpleasant as neutral threat cues.

#### Arousal Ratings

No interaction Instruction × Block × Group was found for arousal ratings (*F*(1,38) = 0.32, *p* = 0.58, η_p_^2^ < 0.01, BF_incl_ = 0.12) indicating the null hypothesis is at least 8.4 times more probable than the alternative hypothesis (1/BF_incl_). Follow-up tests for the angry-neutral group (see Figure 2C) revealed that facial expressions were more arousing when cueing threat compared to safety (*F*(1,19) = 28.29, *p* < 0.001, η_p_^2^ = 0.60). Neither the main effect Block (*F*(1,19) = 0.13, *p* = 0.72, η_p_^2^ < 0.01) nor the interaction Instruction × Block reached significance (*F*(1,19) = 3.25, *p* = 0.087, η_p_^2^ = 0.15). Similarly, for the happy-neutral group, instructed threat cues were more arousing compared to safety cues (*F*(1,19) = 32.79, *p* < 0.001, η_p_^2^ = 0.63), and no differences were observed across Blocks (*F*(1,19) = 3.53, *p* = 0.076, η_p_^2^ = 0.16). The interaction Instruction × Block missed significance (*F*(1,19) = 4.06, *p* = 0.058, η_p_^2^ = 0.18). Thus, a pattern of threat-enhanced arousal ratings (relative to safety) was observed, regardless of facial expression (happy, neutral, or angry faces) and the experimental order of conditions.

### Startle Reflex

**FIGURE 3:**
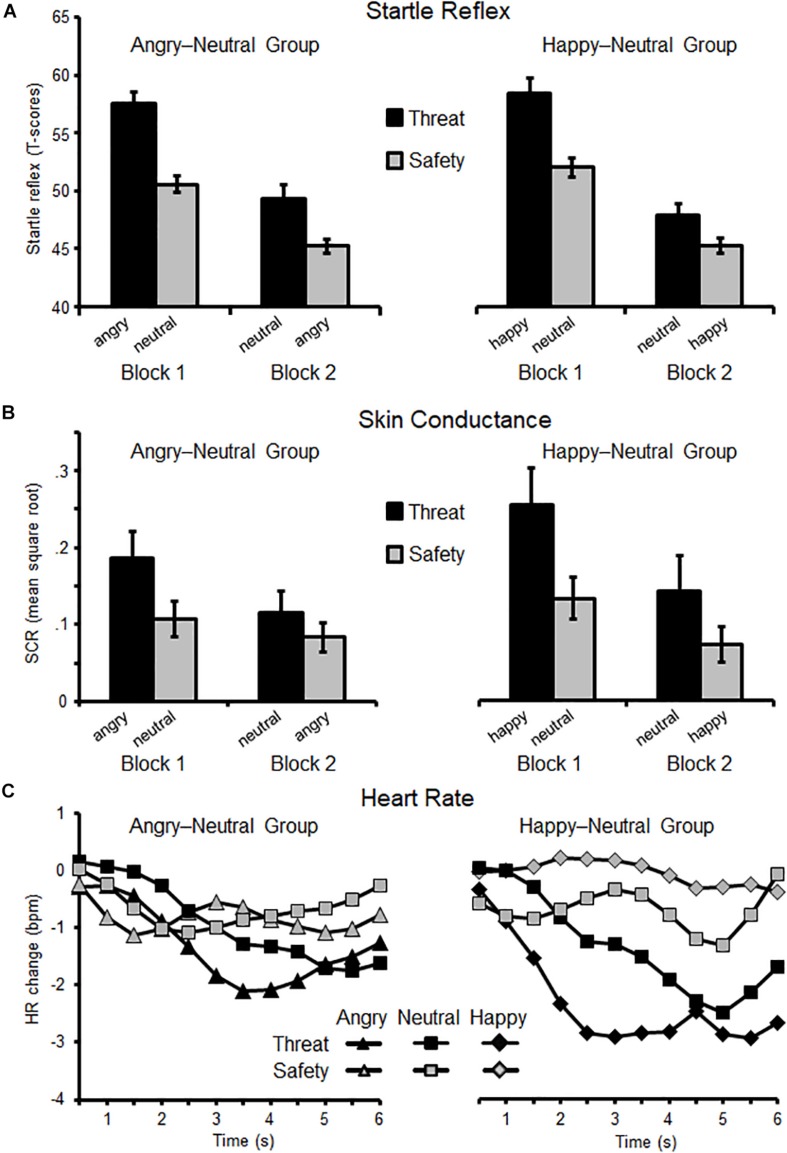
Eye-blink startle reflex **(A)**, skin conductance responses **(B)**, and changes in heart rate **(C)** as a function of Block (first, second) and Instruction (threat, safety). Means (SEM) are plotted separately for each group: on the left side, the Angry–Neutral Group started with angry expression as threat cue, and on the right the Happy–Neutral Group with happy faces cueing threat in the first block. For both groups neutral faces served as reversed threat cue in the second block.

For the defensive startle reflex, the intrinsic emotional valence of an angry or happy facial expression did not modulate the instantiation or reversal of threat effects, Group × Instruction × Block (*F*(1,36) = 0.12, *p* = 0.74, η_p_^2^ < 0.01, BF_incl_ = 0.13) with the null hypothesis being 7.69 times more likely than the alternative hypothesis. Overall, reflex amplitudes were potentiated for threat compared to safety cues (*F*(1,37) = 41.69, *p* < 0.001, η_p_^2^ = 0.53) and decreased across blocks (*F*(1,37) = 157.74, *p* < 0.001, η_p_^2^ = 0.81). Moreover, startle responses varied as a function of Instruction × Block (*F*(1,37) = 4.99, *p* < 0.05, η_p_^2^ = 0.12). Threat effects were significant in both blocks (all *ps* < 0.001) but more pronounced in the first than in the second block. Because we *a priori* predicted facial emotions to modulate reversal learning, follow-up comparisons tested each threat-reversal combination separately (angry-neutral vs. happy-neutral group; see Figure 3A).

When angry faces served as instructed threat cues (Block 1), and then as reversed safety cues (Block 2), main effects of Instruction and Block emerged (*Fs*(1,19) = 38.52 and 61.73, *ps* < 0.001, η_p_^2^ = 0.67 and.77). The interaction Instruction × Block was not significant (*F*(1,19) = 1.62, *p* = 0.22, η_p_^2^ = 0.08) indicating similarly pronounced threat-effects for angry and neutral faces cueing threat in both experimental blocks (all *ps* < 0.01). Regarding the happy-neutral group, main effects of Instruction and Block were observed (*Fs*(1,17) = 11.34 and 111.32, *ps* < 0.001, η_p_^2^ = 0.40 and.87) and the interaction missed significance (*F*(1,17) = 3.77, *p* = 0.069, η_p_^2^ = 0.18). Thus, for the instantiation and reversal of threat associations similarly pronounced threat-potentiated startle reflex was observed regardless of facial expressions.

### Skin Conductance Responses

The *a priori* valence of happy and angry facial expressions did not modulate skin conductance responses during instantiation and reversal of threat (Group × Instruction × Block, *F*(1,36) = 0.02, *p* = 0.97, η_p_^2^ < 0.01, BF_incl_ = 0.072), suggesting the null hypothesis is 13.89 times more probable relative to the alternative hypothesis. Regardless of facial expressions, SCRs were more pronounced for faces cueing threat compared to safety (Instruction *F*(1,36) = 13.66, *p* = 0.001, η_p_^2^ = 0.28), and diminished across experimental blocks (Block *F*(1,36) = 11.33, *p* < 0.01, η_p_^2^ = 0.24). A significant interaction of Instruction × Block (*F*(1,36) = 4.78, *p* < 0.05, η_p_^2^ = 0.12) indicated pronounced threat-effects in the first block (*p* < 0.001) and less pronounced but significant in the second block of the experiment (*p* < 0.05). Follow-up comparisons tested each threat-reversal group separately (see Figure 3B).

For the angry–neutral group, when angry faces cued threat in Block 1 (and safety in Block 2), SCRs did not reach a significant level for the main effects Instruction and Block (*Fs*(1,19) = 3.83 and 2.83, *ps* = 0.065 and.11, η_p_^2^ = 0.17 and 0.13). Also the interaction Instruction × Block was not significant (*F*(1,19) = 2.77, *p* = 0.11, η_p_^2^ = 0.13). Similarly, for the happy–neutral group, when happy faces served as initial threat cue (Block 1), and following as safety cue (Block 2), no interaction Instruction × Block was found (*F*(1,17) = 2.07, *p* = 0.17, η_p_^2^ = 0.11).

### Phasic Heart Rate Changes

Heart rate deceleration was more pronounced when viewing threat relative to safety cues (*F*(1,36) = 7.25, *p* = 0.011, η_p_^2^ = 0.17). Furthermore, the interaction of Instruction × Time was significant (*F*(11,396) = 8.43, *p* < 0.001, η_p_^2^ = 0.19) showing significant deceleration for threat relative to safety cues between 2.5 and 6 s after cue onset (all *ps* < 0.05). Neither the main effect Block (*F*(1,36) = 2.99, *p* = 0.092, η_p_^2^ = 0.08) nor the interactions Instruction × Block (*F*(1,36) = 0.42, *p* = 0.52, η_p_^2^ = 0.01), Time × Instruction × Block (*F*(11,396) = 0.35, *p* = 0.78, η_p_^2^ = 0.01) reached significance. The interaction Group × Instruction × Block was not significant (*F*(1,35) = 0.24, *p* = 0.63, η_p_^2^ < 0.01, BF_incl_ = 0.027), suggesting the null (relative to the alternative) hypothesis as 37 times more likely with the given data set.

Follow-up comparisons focused separately on the angry-neutral and happy-neutral groups (see Figure 3C). When angry faces initially served as a threat cue (angry-neutral group), no threat-deceleration was observed (*F*(1,18) = 0.70, *p* = 0.42, η_p_^2^ = 0.04) neither in the first nor in the second Block (*ps* = 0.19 and.91). In contrast, for the happy-neutral group, a pronounced heart rate deceleration was found for instructed threat cues (*F*(1,17) = 9.39, *p* < 0.01, η_p_^2^ = 0.36). Moreover, the interaction Instruction by Time reached significance (*F*(11,187) = 7.23, *p* < 0.001, η_p_^2^ = 0.30) indicating pronounced deceleration for threat relative to safety cues in the time window from 2 to 6 s (all *ps* < 0.05).

### Correlational Analyses

Building upon a previous study (Bublatzky et al., 2018), we examined whether interindividual differences in trait and social anxiety modulated threat-safety reversal learning as indicated by the startle reflex. Whereas no covariations were observed with social anxiety scores (*Fs*(1,35) < 2.50, *p* > 0.12, η_p_^2^ < 0.07), a significant interaction emerged for Instruction by trait-anxiety (*F*(1,35) = 8.20, *p* < 0.01, η_p_^2^ = 0.19). Follow-up analyses revealed a correlation between trait anxiety and threat effects in the first block (*r* = −0.40, *p* < 0.05) indicating that high (relative to low) trait anxious participants differentiated less between threat and safety cues during Block 1, but not in Block 2 (*r* = −0.16, *p* = 0.33).

## Discussion

The present study examined facial emotions as cues for instructed threat or safety. Moreover, reversal instructions served to investigate the malleability of affective associations by means of social learning. Viewing threat cues led to priming of defensive motor reflexes and pronounced activation of the autonomous nervous system. Importantly, physiological reactions to threat cues emerged regardless of whether angry or happy facial expressions signaled shocks. Self-report data, however, revealed interaction effects of visual and verbal information. Angry faces serving as threat cues were perceived as more threatening and unpleasant relative to neutral safety cues, this was not observed for happy faces cueing threat. Regarding reversal learning, verbal instructions were highly effective in changing previously learned affective associations from threat to safety and vice versa. Again, self-reported threat and valence, but not physiological measures, revealed interaction effects between instructed threat and facial expressions. Angry faces maintained their threatening value even when instructed as safety cue, and happy facial expressions tended to be more resistant becoming a threat cue. Thus, the incongruity of intrinsic facial valence and explicitly instructed threat seems to play a role for the conscious perception (i.e., ratings), but not for the activation of the autonomic and somatic nervous systems.

When facial emotions cued threat in Block 1, pronounced activation of physiological response systems were observed (i.e., threat-potentiated startle reflex, enhanced skin conductance responses), which has been suggested to reflect neurobiological defense preparation (e.g., Grillon et al., 1991; Bradley et al., 2005; Bublatzky et al., 2013). For this defense activation, however, the intrinsic valence of facial cues (happy or angry expressions) did not modulate the instantiation of threat responses. This finding provides a direct replication of our recent study showing similarly pronounced instructed threat effects to happy and angry facial cues (Bublatzky et al., 2018). Moreover, results are in line with previous research using affective picture materials as instructed threat cues (e.g., pleasant and unpleasant scenes; Bradley et al., 2005; Bublatzky and Schupp, 2012), showing a flexible change of the intrinsic facial valence and according physiological reactions. Thus, language information can readily overwrite the emotional impact of affective scenes and faces. With regard to face and person perception, this appears highly adaptive as facial expressions are subject to voluntary control and social norms (e.g., Zaalberg et al., 2004; Mallan et al., 2013). Future research might investigate whether invariant facial features, such as identity information or the color of the skin (e.g., structural features; Kaufmann and Schweinberger, 2004; Calder and Young, 2005; Guerra et al., 2012; Golkar and Olsson, 2017), are the more persistent threat or safety cues in person perception.

Key hypotheses concerned the reversal of threat and safety associations across different facial expressions. Shifting threat from one stimulus to another presumably reflects the concurrent acquisition and inhibition of (new and old) threat associations (Schiller and Delgado, 2010). Similar to recent research using abstract objects (e.g., Costa et al., 2015; Mertens and De Houwer, 2016), in the present study verbal instructions were highly effective in changing previously learned threat/safety linked to other peoples’ facial expressions. Interestingly, this reversal process did not vary as a higher-order function of instructed threat, experimental block and/or order (i.e., coding facial expression), for none of the physiological measures. Precluding direct comparisons of angry and happy facial expression on reversal learning (no overall interaction effects for physiological data), results do not support the involvement of prepared learning mechanisms in the instantiation or reversal of threat and safety associations. Specifically, neither angry facial expressions served as a better threat-cue (cf. anger-superiority; Seligman, 1971; Öhman and Mineka, 2001), nor did happy faces more readily acquire safety-qualities (cf. prepared safety signals; Hornstein and Eisenberger, 2018). Using a verbal learning approach, the present findings contribute to the mixed evidence of whether facial information serve as an evolutionary prepared conditioned stimulus.

Here, another noteworthy finding relates to the dissociation of physiological reactions to threat/safety cues and their self-reported evaluations. Neither the somatic reflex system (startle responses) nor the autonomic nervous system (SCR and HR) revealed interactions of facial expressions and instructed contingencies for the instantiation and reversal of threat. However, self-report data indicated that happy faces seemed more shielded to become threatening and unpleasant even when cueing shocks (Block 1). In contrast, angry faces maintained being perceived as threatening and unpleasant despite cueing safety after reversal instructions (Block 2). This result pattern resembles findings from instructed extinction studies (Luck and Lipp, 2015, 2016), showing persistent negative cue evaluations after extinction instructions (but no longer threat-specific physiological responding). Similarly, threat ratings have been observed to be surprisingly stable even without aversive reinforcement across repeated test sessions/days (Bublatzky et al., 2013, 2014a). Seen from a clinical perspective, these findings suggest that physiological indices of threat learning might be more sensitive to cognitive interventions (e.g., safety instructions), and that reducing the perceived negative valence of threat cues requires extended exposure training to prevent relapse of fear (Craske et al., 2008; Luck and Lipp, 2015). Examining the involved mechanisms of social safety learning (based on instructions or observing others; Olsson and Phelps, 2007; Askew et al., 2016) appears particular helpful to improve cognitive-behavioral treatments to overcome the many fears and anxieties that rely on aversive anticipations rather than first-hand experiences.

Several aspects of the present study and experimental design need to be considered. Happy, neutral, and angry facial expressions were presented in both blocks, however, only those expressions that were explicitly instructed as threat or safety cues contributed to the statistical design. Thus, depending on the group, the presence of a non-threatening angry or happy face may have modulated the initial acquisition of threat/safety contingencies. Moreover, the combined use of female and male faces displaying emotional expressions may have modulated the impact of threat/safety instructions (Mazurski et al., 1996) as well as emotional facial expressions on the startle modulation (Hess et al., 2007; Anokhin and Golosheykin, 2010; Paulus et al., 2014). However, the number of startle trials per condition (four) and the imbalance among female and male participants (35:5) precludes testing stimulus and/or participants’ gender as additional statistical factors. Finally, accounting for the clinical relevance of the threat/safety reversal manipulation, we could not replicate a previous study that showed threat effects as more persistent in socially and trait anxious participants (cf. Bublatzky et al., 2018). Instead, more trait-anxious participants tended to differentiate less between threat and safety cues during the instantiation of threat associations (Block 1). Thus, pointing to the importance of interindividual differences, the inclusion of selected high-/low anxious participants and/or patients suffering from anticipatory anxiety is recommended (e.g., social or generalized anxiety disorder). In the same vein, the impact of interpersonal factors such as gender, ethnicity, and social group biases on social threat and safety learning is yet under-explored (e.g., Navarette et al., 2009; Golkar and Olsson, 2017), especially with regard to psychopathology. Here, variations of the expected likelihood of the anticipated event, online ratings of threat expectancy, and repeated reversal instructions may be particular informative (Lovibond and Shanks, 2002; Atlas et al., 2016; Atlas and Phelps, 2018).

In summary, viewing facial emotions, which were instructed to signal threat, triggered pronounced physiological defense preparation. The intrinsic valence of threat cues (happy or angry facial expression), however, did neither modulate the instantiation nor the reversal of threat and safety associations on the physiological level. A different picture emerged for affective ratings: Happy facial expressions tended to be more resistant becoming a threat cue, and angry faces remained threatening even when instructed as safety cue. Thus, the incongruity of visual and verbal threat/safety information modulates conscious perception, but not the activation of physiological response systems. In person perception, language information readily overwrites the intrinsic affective impact of facial emotions. This has clear benefits for social communication as the anticipation of threat and safety readjusts and accurately tracks environmental changes.

## Data Availability

The datasets generated and analyzed during this study are available on request to the corresponding author.

## Ethics Statement

All participants provided informed consent to the study procedure, which was approved by the local ethics committee (University of Mannheim).

## Author Contributions

FB and PG conceived the study. FB was involved in the data collection and drafted the manuscript. FB, MR, and PG contributed to the data analyses and manuscript revision, and read and approved the submitted version.

## Conflict of Interest Statement

The authors declare that the research was conducted in the absence of any commercial or financial relationships that could be construed as a potential conflict of interest.

## Footnotes

1 Groups did not differ regarding age or questionnaire scores, *ts*(38) < 1.29, *ps* > 0.20.

2 KDEF identifiers of eight female (af01, af07, af09, af11, af19, af20, af22, af29), and eight male actors (am02, am03, am07, am08, am10, am13, am14, am25) used in the experiment; face pictures of two additional actors were presented during practice trials (af02 and am11).

3 Please note, depending on the experimental group, either happy or angry faces were excluded from the analysis. For instance, in the angry-neutral group, happy faces never served as a threat cue and thus were omitted from the safety cue analyses to adjust the statistical design. Thus, the comparison of threat and safety condition was based on an equal number of trials.
